# First results of the double-blind randomized placebo-controlled multicenter clinical trial of DIM-based therapy designed as personalized approach to reverse prostatic intraepithelial neoplasia (PIN)

**DOI:** 10.1186/s13167-016-0057-3

**Published:** 2016-04-02

**Authors:** Mikhail Paltsev, Vsevolod Kiselev, Vadim Drukh, Ekaterina Muyzhnek, Igor Kuznetsov, Evgeniya Andrianova, Pavel Baranovskiy

**Affiliations:** 1 National Research Centre (NRC “Kurchatov Institute”), 1, Akademika Kurchatova Pl., Moscow, 123182 Russia; 2 Peoples’ Friendship University of Russia, Miklukho-Maklaya St., 6, Moscow, 117198 Russia; 3 MiraxBioPharma, Closed Joint Stock Company, 12 Kutuzovsky av., 121248 Moscow, Russia; 4 IlmixGroup, Closed Joint Stock Company, 12 Kutuzovsky av., 121248 Moscow, Russia

**Keywords:** 3,3'-Diindolylmethane (DIM), Prostatic intraepithelial neoplasia (PIN), Clinical study, Molecularly targeted treatment, Targeted prevention, Personalized medicine

## Abstract

**Background:**

Targeted pharmacological correction is used extensively in medical practice today. 3,3'-Diindolylmethane (DIM) is known as a substance with various anticancer properties. An interim study of the efficacy of a new drug Infemin on the basis of diindolylmethane (DIM) with improved bioavalability has been conducted.

**Methods:**

The clinical trial had a multicenter, randomized, placebo-controlled, double-blind design and was carried out in two parallel groups. The interim analysis of data included 21 patients diagnosed with a high-grade prostatic intraepithelial neoplasia (PIN). Group 1 (11 patients) received Infemin in a dose of 900 mg of DIM a day, and group 2 (10 patients) received placebo. To assess the efficacy of therapy, the analysis of morphological index (MI) changes based on the results of histological examinations of prostate biopsy specimens was performed, and a proportion of patients with persistent PIN in 12 months after Infemin initiation was calculated. Researchers also evaluated prostate size, urodynamic parameters (Qmax, Qave, Vres), IPSS, and QoL (quality of life) indices and International Index of Erectile Function (IIEF) at 3, 6, 9, and 12 months after the Infemin administration start.

**Results:**

After 12 months of treatment in the Infemin group, MI decreased from 0.50 to 0.08, while in the placebo group, it increased from 0.27 to 0.58; the difference between the groups was statistically significant (*p* = 0.0003, Mann-Whitney test). In 45.5 % of patients in the Infemin group, a complete regression of PIN was also observed, while in the placebo group, PIN regression was not observed in any patients (*p* = 0.053, Yates’ corrected chi-square). Study results in the Infemin group show improvement of maximal urinary flow rate Qmax (53.3 % increase compared to the initial value); however, the statistical significance was not reached (*p* = 0.180, Mann-Whitney test) due to the small sample size. Evaluation of other urodynamic parameters, prostate volume, quality of life, symptoms reflecting urination disorder, and erectile dysfunction symptoms did not reveal significant differences between the Infemin and placebo groups either which is probably due to the small sample size.

**Conclusions:**

The intermediate results of the 21 patients in this multicenter, randomized, placebo-controlled, double-blind study show that Infemin may be a promising drug candidate in patients with  high-grade PIN.

**Trial registration:**

www.chictr.org.cn ChiCTR-INR-15007496

## Background

Prostate cancer (PC) remains one of the most common oncological problem in developed countries [1]. In 2012, 1.1 million new cases of PC and 307 thousand deaths from this disease were recorded worldwide [2]. In Russia in 2013, about 31.5 thousand new cases of PC were diagnosed; at that, morbidity and mortality per 100 thousand of male population were 47.51 and 16.72, respectively [3].

Prostatic intraepithelial neoplasia (PIN) is considered a morphological equivalent of prostate precancer. It develops as a result of proliferative changes of ductal epithelium and acini of the prostate [4]. Many researchers distinguish two forms of PIN: low-grade PIN (low-grade prostatic intraepithelial neoplasia) and high-grade PIN (high-grade prostatic intraepithelial neoplasia) depending on pronouncement of cytological and structural changes of the epithelium lining the prostate [4]. Nowadays, diagnosis of PIN is usually made when changes specific to high-grade PIN only are revealed since changes specific to low-grade PIN are difficult to distinguish from the normal tissue and/or atypical hyperplasia [5, 6]. Different researchers reveal PIN as a disease predisposing PC in 38–100 % of patients with a confirmed diagnosis of high-grade PIN [7].

It is generally recognized that the development of hyperplastic processes in the prostate is primarily associated with hormonal imbalance [8]. With age in the prostate, increase of enzyme 5-α-reductase production is observed; this enzyme is responsible for synthesis of hormone 5α-dihydrotestosterone (DHT), the active metabolite of male sex hormone testosterone [9]. DHT with its increased androgenic activity leads to the increased activity of genes responsible for proliferation of prostate cells and, consequently, to the development of hyperplasia.

Male sex hormones are known to manifest their biological activity via androgen receptors (ARs). Nevertheless, blocking of hormonal stimuli (surgically or pharmacologically) does not always suppress the development of pathological processes in the prostate. Many molecular cell mechanisms have recently been revealed, stimulation of which leads to the ARs biological function disturbance and, consequently, to their abnormal activation by low levels of androgens or other non-hormonal inducers and, finally, to the development of androgen-refractory PC [10, 11]. Thus, it is becoming clearer that the adequate management of proliferative diseases of the prostate must involve not only androgen-dependent but also other androgen-independent elements of the pathogenesis.

Moreover, with age increase of the level of female hormones, estrogens in men is observed. Estrogens stimulate stromal cells of the prostate via estrogen receptors as well as influence ductal cells sensitive to estrogens which results in abnormal cell proliferation and inflammation [12], mechanisms playing an important part in the PC pathogenesis [13].

Finally, malignant transformation of the prostatic cells is accompanied by the epigenetic regulation disturbance, particularly the increase of DNA methylation in promoter regions and deacetylation of chromatin histones resulting in epigenetic suppression of tumor suppressor genes [14, 15].

For prevention of PC in patients at risk of this disease, a search of pharmacological substances is being conducted which would affect different elements of PIN pathogenesis and allow suppression of transformation of the prostatic cells into tumor cells.

An active substance indole-3-carbinol (I3C) and its physiological metabolite 3,3′-diindolylmethane (DIM) are compounds with confirmed multiple antitumor activity [16]. I3C and DIM are reported to inhibit growth of androgen-dependent and androgen-independent prostatic cell cultures/tumors in vitro and in vivo [17, 18] due to normalization of sex hormone level as well as balanced regulation of androgen and estrogen receptor activity [19]. DIM is proved to be able to reactivate functioning of tumor suppressor genes due to its DNA-demethylating activity as well as its ability to inhibit activity of enzyme histone deacetylases [20, 21]. Moreover, DIM promotes interferon system activation, particularly IFN-γ [22], has a strong anti-inflammatory activity [23], suppresses angiogenesis factor VEGF activity, and markedly decreases metastatic potential of cells acting via a wide range of appropriate molecular targets [24, 25]. Finally, DIM manifests selective activity towards a pool of so called cancer stem cells which are currently considered the main source of tumor recurrences and metastases [26].

In order to improve bioavailability of DIM, a new drug Infemin was developed which constitutes a solution of DIM and excipients into hard gelatin capsules [27].

We have conducted an interim analysis of the efficacy data obtained from double-blind placebo-controlled clinical study (phase IIa) of the efficacy of a new formulation of DIM in patients with high-grade PIN. This article adheres to CONSORT guidance for clinical trials reports.

## Methods

### Study design

The current clinical trial had a multicenter, randomized, placebo-controlled, double-blind design and was carried out in two equal parallel groups. The trial was conducted in 18 study sites located in the Russian Federation. The trial was approved by the Ministry of Health (resolution number 779 dated 24.12.2013, http://grls.rosminzdrav.ru/CIPermitionReg.aspx) and local Ethics Committees of the study sites. The study included a total of 120 patients with the following eligibility criteria.

The inclusion criteria were as follows: written informed consent to participate in this study; diagnosis of prostatic intraepithelial neoplasia (PIN), histologically verified in central reference laboratory; age of 18–80 years; ability to carry out the procedures according to the trial protocol; no official or other forms of relations to the persons involved in the study interested in its outcomes; residual urine volume ≤150 ml, PSA level ≤10 ng/ml.

The exclusion criteria were as follows: history of surgical interventions on pelvic organs or their planning in the nearest 12 months; maximal urinary flow rate <5 ml/s; treatment of chronic prostatitis within 1 month before the first study dose and treatment of prostatic hyperplasia or PIN within 3 months before the first study dose; alcohol or drug abuse; mental disorder and/or uncontrolled physical conditions; use of other investigational medicines within 30 days before the first study dose; prostate cancer and other malignant neoplasms; acute urine retention; neurogenic dysfunctions and bladder ears; urethral stricture; bladder neck sclerosis; urinary infections in a phase of active inflammation; bladder calculi; diseases of cardiovascular and nervous system, concomitant renal or hepatic failure; positive hepatitis B or C, syphilis, or HIV tests.

Evaluation of the efficacy of therapy was performed based on the changes of morphological index (MI) by the moment of therapy completion (a statistically significant decrease of MI compared to placebo). MI was developed by us in order to quantify neoplastic changes in prostate and was calculated according to the formula:

$$ \mathrm{M}\mathrm{I}=\left\{\left[\mathrm{number}\ \mathrm{of}\ \mathrm{low}\hbox{-} \mathrm{grade}\ \mathrm{PIN}\ \mathrm{foci}\right]+2*\left[\mathrm{number}\ \mathrm{of}\ \mathrm{high}\hbox{-} \mathrm{grade}\ \mathrm{PIN}\ \mathrm{foci}\right]+3*\left[\mathrm{number}\ \mathrm{of}\ \mathrm{cancerous}\ \mathrm{foci}\right]\right\}/\left[\mathrm{number}\ \mathrm{of}\ \mathrm{biopsy}\ \mathrm{fragments}\right] $$

We developed this index as a measure of both severity of neoplastic changes and their volume. The number of biopsy fragments is used in MI formula in order to prevent bias in cases when more or less than 12 biopsy cores were taken.

The study’s hypothesis on the effectiveness of the drug is that its use should lead to a difference in MI compared to a placebo. Thus, if the MI by the end of the study in the placebo group and in study group is equal to h0 and h1, respectively, null and alternative hypotheses can be formulated as follows:

- H0: h0 = h1, there are no treatment-dependеnt differences in the MI.
- H1: h0 ≠ h1, there are treatment-dependеnt differences in the MI.

The need to verify the hypothesis of the study (MI’s difference between groups) was used as a basis in order to perform calculations of the proper sample size. Study statistical power was taken as 90 % (*β* = 0.1) and the significance level (*α*) was taken as 0.05 (two-tailed). As it was planned to perform two Holm-adjusted comparisons (interim and final), the sample size was calculated according to the lowest *α*/2 level = 0.025. It was decided that the hypothesized MI in study groups may reach 0.7 ± 0.3; for the purposes of calculating the effectiveness in the placebo group, this was taken as 0.9 ± 0.3 (on the basis of expert opinions). For *β* = 0.10 and *α* = 0.025, with the assumed difference between the MIs, we needed 58 subjects with available data for analysis in the each group. This value was increased to 60 assuming 3 % patient dropout rate.

Calculation of the sample size was performed as follows:

$$ n = {\left[{Z}_{\alpha }+{Z}_{\beta}\right]}^2*\ \left({S_{\mathrm{xe}}}^2+{S_{\mathrm{xc}}}^2\right)/{\delta}^2, $$

where *n* is the sample size for each group, *S*_xe_ is the standard deviation in the first group, *S*_xc_ is the standard deviation in the second group, *δ* is the difference between groups’ MIs, and *Z*_*α*_ and *Z*_*β*_ are the critical values of normal distribution corresponding to a given level of errors type 1 and 2.

**Fig. 1:**
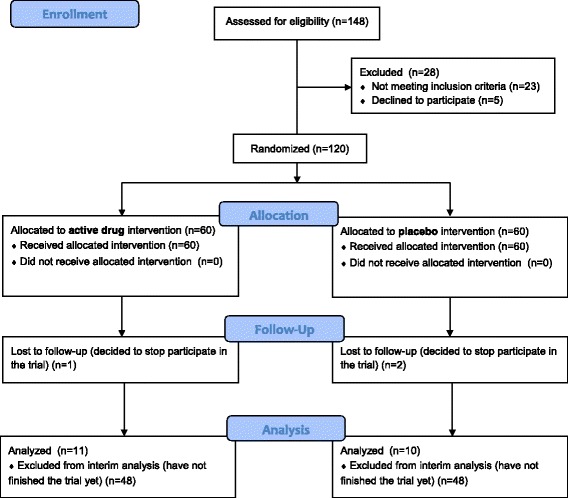
Patient flowchart to the moment of current interim analysis

A randomization list was provided by the sponsor before the beginning of the study using SPSS Statistics version 20.0.0 computer software. Block randomization was used with a block size equal to 2, each block containing one patient who was assigned an active drug and one patient who was assigned a placebo. The blinded treatment assignment procedure was carried out by sending a fax to the sponsor from the study site. The fax contained a randomization request form with information on the patient’s conformity with the inclusion/exclusion criteria. The sponsor’s response, sent by the fax to the study site, consisted of a patient report form with a unique identification number and drug identification code on the packaging (in accordance with the randomization sheet, packages of the preparation contained an appropriate daily dose of the active drug or a placebo). The randomization sheet was kept solely by the sponsor. Patient flow diagram is shown in Fig. 1.

### Patients and treatment

Twenty-one patients, age 52–78 years old, with histologically verified diagnosis of high-grade prostatic intraepithelial neoplasia (PIN) were included in current interim analysis as they have completed the trial. Initially, all the patients had a residual urine volume ≤150 ml, PSA level ≤10 ng/ml, and maximal urinary flow rate ≥5 ml/s.

Twenty-eight days before the active therapy, patients underwent screening during which their medical history was taken; physical examination and laboratory test were performed. Laboratory tests included complete blood count, clinical urine test, blood chemistry (general protein, glucose, creatinine, general bilirubin, aspartate aminotransferase (AST) and alanine transaminase (ALT) activity), and serum PSA level. Also, hepatitis B and C and HIV and RW blood diagnostics were performed.

In the course of initial screening, ECG data (PQ, QRS, QT) of patients were obtained, and urological checkup with digital rectal examination, prostate biopsy, uroflowmetry, transrectal ultrasonography (TRUS) with retained urine determining, and completion of questionnaires (IPSS + QoL, IIEF) were performed.

After signing the informed consent and checking of eligibility criteria, the participants were divided into two groups. Recruitment began on 30 January 2014 and has been finished on 28 July 2015, the day when the 120th patient was randomized. Of the total of 120 patients, three patients (one in active drug group and two in placebo group) were lost to follow-up because they decided to stop participating in the trial (to the moment of interim analysis). Twenty-one patients who completed the study to the moment were included in the current interim analysis. Patients of group 1 (11 patients) were prescribed with Infemin in the initial dose of 900 mg of DIM a day (three capsules two times a day); group 2 (10 patients) received placebo (three capsules two times a day). Active therapy was performed for 12 months with control visits at the beginning of study, in 3, 6, and 9 months after the treatment start.

Ultrasonography-guided 12-core prostate biopsy was performed for every patient during the screening and at the end of study (after 12 months of treatment). In cases of significant PSA increase and clinical symptoms, progression biopsy might be performed at 3, 6, or 9 months after the trial start too. Tissue fragments were taken from both prostate lobes (six from the left and right). Specimens were fixed in 10 % buffered formalin solution and then embedded in paraffin. Sections were stained with hematoxylin and eosin for routine histological examination, as well as for immunohistochemical studies (when needed) in a central reference laboratory.

A proportion of the patients with persistent PIN/BPH in 12 months after the Infemin therapy start and a proportion of patients with PC in 12 months after the Infemin therapy start were used as additional efficacy criteria. Researchers also determined (1) prostate size (mL) in 3, 6, 9, and 12 months after the Infemin administration start (using the formula [width × height × length] × 0.70); (2) urodynamic parameters: maximal urinary flow rate (Qmax), average urinary flow rate (Qave), residual urine volume (Vres) in 3, 6, 9, and 12 months after the Infemin administration start; and (3) general IPSS indixes (symptom intensity), QoL (quality of life), International Index of Erectile Function (IIEF), and IEEF subindices (A, B, C, D, E) in 3, 6, 9, and 12 months after the Infemin administration start. Analysis of the efficacy parameters was performed based on the results of histological examinations of prostate biopsy specimens and TRUS, urodynamic parameters and data of IPSS, and QoL and IIEF questionnaires.

### Test compositions

Infemin capsules (“IlmixGroup,” Closed Joint Stock Company, Russia) contain DIM (150 mg), cod liver oil (20 mg), α-tocopherol acetate (5 mg), and polysorbate 80 as an excipient (575 mg). Comparator drug—placebo, contains polysorbate 80 (750 mg). Active drug and placebo had the same organoleptic characteristics.

### Statistical analysis

All calculated parameters are expressed as Me (Q1; Q3), where Me is median and (Q1; Q3) is interquartile range: upper limit of lower quartile (Q1) and lower limit of upper quartile (Q3). As the number of patients was too low to use parametric methods, to determine statistical significance of differences between groups, Mann-Whitney *U* test and Yates’ corrected chi-square (for binary variables) were used. Differences in current interim analysis were considered statistically significant when *р* < 0.025 according to Holm adjustment for multiple comparisons. Statistical analysis of study results was performed using SPSS Statistics 19.0 and Microsoft Excel 2007 software packages.

## Results

The interim analysis included male patients, age 52–78 years old, with verified diagnosis of high-grade PIN. The two study groups (active therapy and control) did not differ from each other in main demographic parameters (race, age, smoking status) and in the initial condition (history of diseases and concomitant diseases) before the inclusion into the study. In study groups, no significant differences in main physical parameters, laboratory parameters of blood and urine, ECG, urodynamic parameters, and PSA level were determined. The groups did not differ in IPSS, QoL, and IIEF indices (general and five subindices A, B, С, D, E). The data obtained suggested the possibility of assembling the patient into a group for analysis considering the study sample to be homogeneous.

Patients in group 1 (11 patients) were prescribed with Infemin orally in the initial dose of 900 mg of DIM a day (three capsules two times a day); patients of group 2 (10 patients) received three capsules of placebo two times a day. The therapy was conducted for 12 months.

**Fig. 2:**
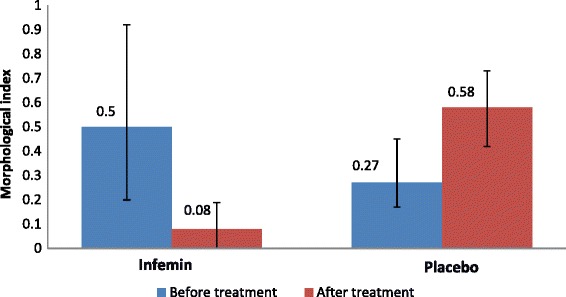
Morphological index value in patients diagnosed with high-grade PIN before and after 12 months of treatment in the Infemin and placebo groups. Data are expressed as Me (Q1; Q3)

Based on the results of the interim data analysis obtained during this randomized double-blind placebo-controlled study of the efficacy of Infemin in treatment of PIN, changes of the main efficacy criterion, morphological index, were assessed (Fig. 2).

It was determined that while before the treatment in the Infemin group MI was 0.50 (Q1 0.20; Q3 0.93), after 12 months of the therapy, MI decreased to 0.08 (Q1 0.00; Q3 0.20). At the same time in the placebo group, an increase of MI from 0.27 (Q1 0.17; Q3 0.44) to 0.58 (Q1 0.42; Q3 0.73) was observed. Statistical analysis in study groups in 12 months showed that in the Infemin group MI was significantly lower (*p* = 0.0003, Mann-Whitney test) compared to the placebo group, 0.08 versus 0.58, respectively.

**Fig. 3:**
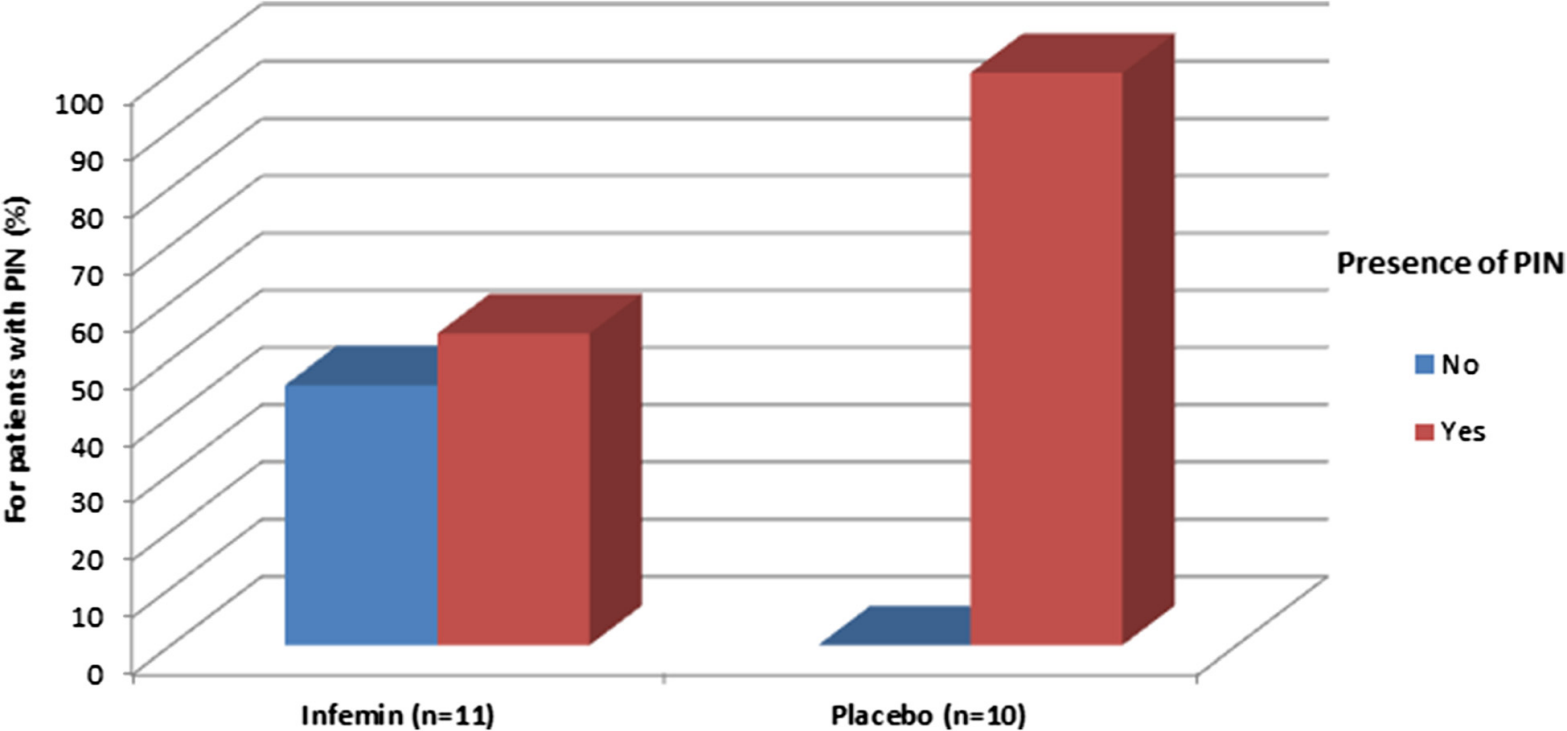
Proportion of patients with persistent high-grade PIN in the Infemin and placebo groups in 12 months after the treatment start

The most important additional efficacy criterion for the treatment of PIN is a complete response rate. In this study, a proportion of patients with persistent high-grade PIN was assessed based on the results of the treatment with Infemin. It was determined that the therapy with Infemin conducted for 12 months resulted in the complete regression of PIN in 45.5 % of patients (five of 11 patients). At the same time, the regression of PIN was not observed in either of patients in the placebo group (Fig. 3). However, with such a sample size, statistical significance of the differences between the Infemin and placebo groups in this parameter was not reached (*p* = 0.053, Yates’ corrected chi-square).

After 12 months of the follow-up, PC was determined in three patients (only in the placebo group −30.0 %). To this moment, the difference in PIN progression to PC rate between groups has not reached statistical significance (*p* = 0.181, Yates’ corrected chi-square).

In the analysis of the additional criteria of the efficacy of therapy, no significant changes (Mann-Whitney test) were observed to the end of follow-up period (Table 1).

**Table 1:** Changes of the treatment efficacy parameters in the Infemin and placebo groups

Parameter	Infemin Me (Q1;Q3)		Placebo Me (Q1;Q3)	
	Screening	12 months	Screening	12 months
Prostate size, mL	52 (37;71)	65 (54;67)	59 (48;75)	42 (20;109)
Maximal urinary flow rate (Qmax), mL/s	10 (7;14)	17 (14;17)	17 (12;19)	15 (15;16)
Average urinary flow rate (Qave), mL/s	6 (3;7)	9 (7;10)	9 (7;11)	10 (8;12)
Residual urine volume (Vres), mL	30 (14;60)	42 (15;65)	23 (18;40)	25 (0;35)
Symptom intensity (IPSS index)	14 (11;20)	4 (2;6)	14 (6;19)	7 (6;9)
Quality of life (QoL index)	3 (3;3)	1 (1;1)	2 (2;5)	2 (2;6)
Erectile dysfunction (IIEF index)	26 (6;43)	51 (46;55)	28 (8;51)	63 (35;65)

Based on the comparison results for the treatment efficacy in the study groups in quality of life (QoL), symptoms reflecting urination disorder (IPSS index), and various aspects of patients’ sexual function (IIEF index and A, B, C, D, E subindices), no significant differences were determined after 12 months of the therapy (Mann-Whitney test). Quantitative values of the investigated parameters are showed in Tables 1 and 2.

**Table 2:** Changes of various aspects of patients’ sexual function (IIEF index and A, B, C, D, E subindices) in the Infemin and placebo groups

Parameter	Infemin Me (Q1;Q3)		Placebo Me (Q1;Q3)	
	Screening	12 months	Screening	12 months
Erectile function itself subindex (A)	12 (4;21)	22 (18;25)	28 (15;29)	6 (1;26)
Orgasmic function subindex (B)	4 (0;10)	7 (6;7)	4 (0;10)	10 (5;10)
Libido intensity subindex (C)	4 (3;6)	7 (6;7)	5 (2;6)	7 (5;8)
Satisfaction with sexual intercourse subindex (D)	6 (0;8)	10 (10;10)	6 (0;9)	11 (5;11)
General satisfaction with sexual function subindex (E)	4 (3;6)	6 (6;6)	5 (2;6)	8 (5;8)

## Discussion

This study represents interim analysis of data obtained in phase IIa clinical trial; the main aim of this trial is a proof of concept. We performed an interim study in order to show whether the investigative drug has a treating potential against PIN. Results of this interim double-blind placebo-controlled study confirm that the administration of Infemin for 12 months in patients diagnosed with high-grade PIN results in marked improvement of the morphological structure of prostate (MI decrease from 0.50 to 0.08) which is indicative of the complete suppression of the proliferative activity of prostatic epithelium induced by this drug. In 45.5 % of patients in the Infemin group, a complete regression of PIN was also observed which is an important additional criterion of the treatment efficacy; however, the significance of the differences was not reached with such a small number of patients. The assessment of urodynamic parameters, prostate volume, quality of life, symptoms reflecting urination disorder, and symptoms of erectile dysfunction did not revealed statistically significant differences compared to the placebo group which is probably due to the insufficient sample size. Improvement of the condition for these parameters will be analyzed for a larger number of patients.

The first data about DIM efficacy in PIN patients are of special interest because there is no cure for this condition at the moment. Very few publications were focused on this problem. The only promising agent known to this time is green tea extract (catechins). It was reported that green tea catechins were effective in high-grade PIN patients [28]. High-grade PIN often leads to prostate cancer, and this group of patients needs effective treatment as a personalized approach for PC risk reduction. Our concept is that PC chemoprevention should be focused at high-grade PIN patients. We are going to present the complete results of this phase IIa trial later, and during the IIb phase, clinical trial dose ranging and pharmacodynamic studies will be performed. Future pharmacoepidemiology studies are needed also to obtain the final proof of this concept.

Novel DIM-based formulation Infemin with high bioavailability may represent effective targeted treatment for patients with high-grade PIN. Many practical applications of DIM are based on its ability to target multiple molecular and biochemical signaling pathways. Therapy with Infemin may become a preventive measure that contributes to the suppression of the pathological processes in prostate at the early stage. The results from the current trial provide preliminary evidence that DIM-based formulation may be a promising medication for individuals at high risk of PC. Previously, we obtained preliminary data indicating a good tolerability profile of Infemin in patients with PIN [29]. We believe that prostate cancer risk might be diminished thanks to a unique therapeutic potential of this active substance.

## Conclusions

The presented results of the efficacy study of the Infemin are intermediate (the study is scheduled to be completed in 2016). The main limitation of this trial is a small number of patients in the interim analysis. However, based on these intermediate results, already obtained for the 21 patients with high-grade prostatic intraepithelial neoplasia, a potentially favorable efficacy profile of the Infemin has been demonstrated. Therefore, it is concluded that Infemin may be a promising drug candidate for the treatment of high-grade PIN and, thus, for the prevention of prostate cancer in these patients. Further clinical studies are needed to detect the efficacy and safety of the drug.

## Abbreviations

ALT: alanine transaminase
AR: androgen receptor
AST: aspartate aminotransferase
BPH: benign prostatic hyperplasia
DHT: 5α-dihydrotestosterone
DIM: 3,3'-diindolylmethane
ECG: electrocardiogram
I3C: indole-3-carbinol
IIEF: International Index of Erectile Function
IPSS: International Prostatic Symptoms Scale
MI: morphological index
PC: prostate cancer
PIN: prostatic intraepithelial neoplasia
PSA: prostate-specific antigen
QoL: quality of life
